# Long-Term *Wu Qin Xi* Exercise on Response Inhibition and Cortical Connectivity in Parkinson's Disease: Design and Implementation of a Randomized Controlled Clinical Trial

**DOI:** 10.3389/fneur.2021.675050

**Published:** 2021-07-19

**Authors:** Zhen Wang, Lan-Lan Zhang, Yin Wu, Jian Zhang, Ke Liu

**Affiliations:** ^1^School of Exercise and Healthy Science, Xi'an Physical Education University, Xi'an, China; ^2^School of Psychology, Shanghai University of Sport, Shanghai, China; ^3^School of Leisure Sport and Management, Guangzhou Sport University, Guangzhou, China; ^4^School of Economics and Management, Shanghai University of Sport, Shanghai, China; ^5^Shanghai Punan Hospital of Pudong New District, Shanghai, China

**Keywords:** Parkinson's disease, *Wu Qin Xi* exercise, response inhibition, cortical connectivity, TMS

## Abstract

**Background:** Motor symptom disorders in patients with Parkinson disease (PD) are closely related to reduced inhibitory ability. Although exercise has been shown to improve this ability in patients with PD, its effects on proactive and reactive inhibition have not been determined. Most previous studies of inhibitory control disorder in people with PD have been behavioral, and little attention has been paid to functional cortical connectivity. *Wu Qin Xi*, a low–medium-intensity *qigong* exercise that is safe and easy to do for elderly individuals, can support physical well-being and help prevent and alleviate disease. In this study, our aims were to explore the effects of a long-term *Wu Qin Xi* intervention on response inhibition and to examine how improved inhibition control relates to cortical connectivity using dual-site paired-pulse transcranial magnetic stimulation (ppTMS), in patients with mild–moderate PD.

**Methods:** A single-blind randomized controlled trial will be conducted. A total of 90 elderly subjects will be recruited and allocated randomly to *Wu Qin Xi*, balance exercise, and healthy control groups. The exercise interventions will be implemented in three 90-min sessions per week for 24 weeks; the healthy control group will receive no intervention. The primary assessments will be response inhibition metrics and task-based ppTMS. The secondary outcomes will include motor symptom severity, mobility, balance, emotional state, and quality of life. Assessments will be conducted at baseline, at the conclusion of the intervention period (week 24), and a few months after the intervention (week 36 follow-up).

**Discussion:** This study is designed to provide insights into the effects of practicing *Wu Qin Xi* on response inhibition function in people with PD. The results will provide evidence on the value of traditional Chinese exercise as a therapeutic rehabilitation option for these patients. They will also provide data addressing how brain function–related cortical connectivity is related to reactive vs. proactive inhibition in people with PD participating in an exercise intervention.

**Clinical Trial Registration:** This study has been registered prospectively in the Chinese Clinical Trial Registry (ChiCTR2000038517, 18 January 2021).

## Introduction

A central part of motor control is response inhibition, defined as the ability to cancel planned movements effectively (1). Inhibitory disorders, manifesting as poor ability to inhibit behaviors in various contexts, are prevalent among people with neurodegenerative diseases, such as Parkinson disease (PD) (2, 3). Response inhibition is classified as reactive or proactive. Reactive response inhibition describes the unexpected inhibition of an anticipated response. An example of reactive inhibition control is when one slows down to await a signal that will tell them what to do. Conversely, proactive response inhibition describes the outright termination of the response process (4). An example of proactive inhibition control is when one is anxiously waiting for a red light to turn green actively planning to immediately step on the gas as soon as it does. Di Caprio et al. (5) documented reactive inhibition deficits in patients with early-stage PD. However, potential effects of PD on proactive inhibition have not been examined. Differentiating between these two control modes in people with PD would be helpful toward obtaining an in-depth understanding of inhibitory control disorders in this population.

The frontal–subcortical network may be important for inhibitory control (6, 7); changes in its structural function leads to cognitive and motor declines (8) and have been reported to be accompanied by a decline in cortical connectivity (9, 10). Clinical studies suggest that the right hemisphere is important for inhibitory function (11). Duchesne et al. (12) showed that improvement of inhibitory ability in people with PD is largely dependent upon the integrity of the prefrontal network. Proactive inhibition and reactive inhibition activate common brain regions in the response inhibition network, including the right inferior frontal gyrus (rIFG), dorsolateral prefrontal cortex (DLPFC), pre-supplementary motor area (pre-SMA), and subthalamic nucleus (STN) (13). Activity in these regions has been associated with stop commands from the STN (in the basal ganglia). The STN inhibits basal ganglia output that has an inhibitory effect on the primary motor cortex (M1), thus intercepting motor behavior. In the case of weak activation (proactive inhibition), the motor output is immobilized; in the case of strong activation (reactive inhibition), it is stopped completely (4). Executive function modulation of downstream activities requires coordinated processes between the prefrontal lobe and associated brain regions (9). The interconnectivity of interior frontal and motor cortices specifically may play a crucial role in successful inhibitory control (13). Ponzo et al. (14) found a weak inhibitory cortico-cortical interaction between the inferior frontal cortex and contralateral motor cortex and obtained data suggesting that it could be involved in levodopa-induced dyskinesia.

The motor symptoms of PD have been related to decreased inhibitory ability (15). Pharmacological treatments are not very effective for alleviating response inhibition deficits, and the long-term use of such treatments may cause impulse control disorder (ICD). Exercise may be used as a supplement to medical interventions, including pharmacotherapy and functional surgery, to improve the quality of life of patients with PD by delaying motor degeneration and extending functional independence (16). As a supplemental treatment, exercise can help reduce symptoms and slow the progression of PD (10). The potential effects of exercise on reactive and proactive inhibition have not yet been investigated in people with PD.

*Wu Qin Xi* is an integrated *qigong* exercise system that consists of movements that imitate the technical actions and breathing of five animals (tiger, deer, bear, monkey, and crane) while attending to unification of the regulation of one's body, respiration, and mind (17). It incorporates movement of all body joints and stretching of the upper and lower limbs and is thus considered to be an effective mind–body rehabilitative intervention for elderly individuals (18). *Wu Qin Xi* has been found to improve hand dexterity in patients with mild–moderate PD (19). Thus, we plan to explore the effects of long-term *Wu Qin Xi* practice on reactive and proactive inhibition and whether it improves the connectivity of brain regions related to inhibitory control in people with PD.

The stop task is becoming a gold standard for the evaluation of inhibition control. In the stop task, stop signal response time (SSRT) is used as a metric of one's ability to respond to stop orders and considered to be an indicator of reactive inhibition efficiency (20). Meanwhile, proactive inhibition is assessed by measuring the effect of stop-signal probability on go-trial response times (13). Although there is relevant evidence to better cognitive control processes (21–23), a causal relationship between cortex function and motor output has not been proven. Due to the limitations of drug and surgical treatment, non-invasive stimulation therapies, including transcranial magnetic stimulation (TMS), have been recognized among clinicians as a potential therapeutic intervention for PD. In addition to altering excitability at the site of stimulation, repeated magnetic pulses have been shown to influence brain regions anatomically connected to the stimulation site (24). TMS has been reported to ameliorate bradykinesia when applied to M1 at high (≥5 Hz) frequencies or when applied to motor regions anterior to M1 low (≤1 Hz) (25). Continuous theta-burst stimulation over the inferior frontal cortex has been shown to restore weak inhibitory cortico-cortical interactions between the inferior frontal and contralateral motor cortex in people with PD (14). Dual-site paired-pulse transcranial magnetic stimulation (ppTMS) can be used to explore interactions between brain functional areas and the motor cortex (26) and to detect the promotion and inhibition of M1 activity, thereby providing physiological evidence for causal relationships of cortico-cortical interactions, including information regarding directionality and timing (27). Such data have been used to detect bilateral excitatory and inhibitory changes in M1 accurately in healthy elderly people (28).

We propose a prospective randomized clinical trial designed to examine the effects of *Wu Qin Xi* training on the primary outcomes of response inhibition ability and cortical connectivity using task-based ppTMS and on the secondary outcomes of balance, emotional status, and quality of life in patients with mild–moderate PD. We hypothesize that *Wu Qin Xi* training will improve response inhibition ability and increase brain network connectivity and response inhibition coherence in the motor network in this population. We further hypothesize that observed network connectivity changes will occur in association with improvements in response performance.

## Methods

### Study Design

**Figure 1:**
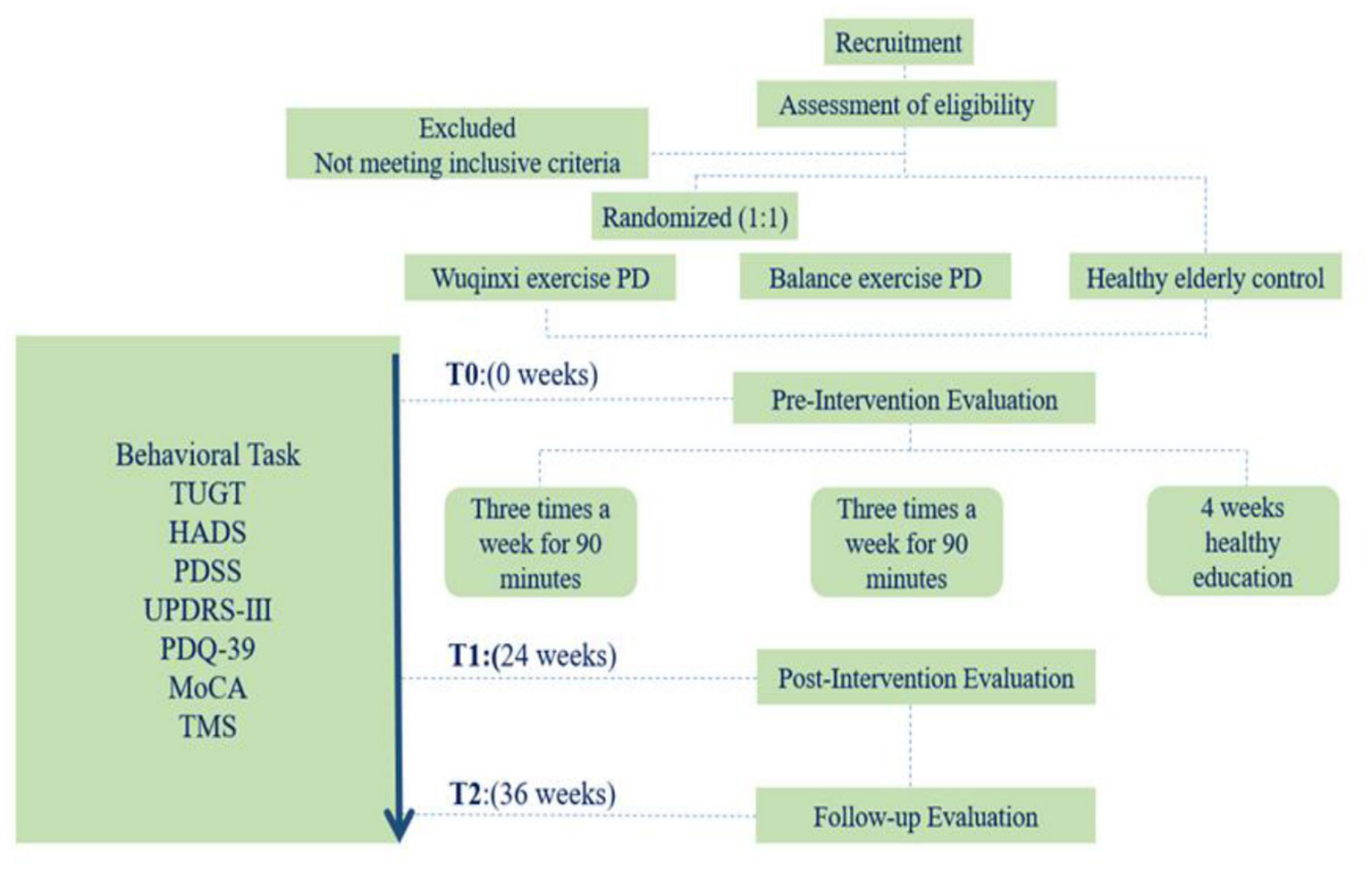
Study flow. Flow diagram of the study design. HADS, Hospital Anxiety and Depression Scale; MoCA, Montreal Cognitive Assessment; PD, Parkinson disease; PDQ-39, 39-item Parkinson Disease Questionnaire; PDSS, Parkinson's Disease Sleep Scale; TMS, transcranial magnetic stimulation; TUGT, timed up-and-go test; UPDRS, Unified Parkinson's Disease Rating Scale Part III.

For this prospective, single-blind, randomized controlled trial, eligible participants with PD will be randomized to *Wu Qin Xi* and balance groups at a 1:1 ratio. Both the *Wu Qin Xi* group and the balance training group will receive group-based exercise intervention at the sports laboratory of Shanghai University of Sport. Participants who meet the criteria will undergo evaluations at baseline, immediately after the intervention, and at 36-week follow-up intervals (Figure 1). This study protocol has been approved by the Shanghai University of Sport Research Ethics Committee (102772020RT107).

### Participants

Potential recruits for this study will be prescreened through outpatient neurology clinics, media advertising, and telephone outreach in the region of Shanghai, China. Participants will be enrolled *via* convenience sampling. To obtain a standard measure of inhibitory control and to enable the examination of differences in reactive and proactive inhibition between healthy elderly individuals and people with PD, we will recruit healthy right-handed elderly participants aged 55–75 with normal or corrected-to-normal vision and no history of neurological disease. TMS will be applied to healthy control participants just as it will be applied to patients with PD.

Participants will follow their regular medication regimens during the study period. All participants will be required to provide written informed consent prior to study inclusion. Detailed information to be collected on participants is listed in Table 1. Information on participants' current medication use, including dosages, will be collected at all evaluations so that changes in medication over the course of the study can be noted and controlled for in statistical analyses. All participants will maintain diaries to record daily exercise, in the laboratory and at home, throughout the trial.

**Table 1:** Demographic and clinical characteristics of the study participants.

		***Wu Qin Xi* exercise PD (*N*)**	**Balance exercise PD (*N*)**	**Healthy elderly (*N*)**
Age, mean (SD), years				
Sex (females), *N*				
Education, years				
Living status	Alone			
	With spouse			
	Family			
MoCA				
HADS				
PDSS				
PDQ-39				
TUG (s)				
Duration of disease, years				
Antiparkinsonian medications	Levodopa or carbidopa			
	Pramipexole or ropinirole			
	Other			
UPDRS-III				
Hoehn & Yahr stage				

*HADS, Hospital Anxiety and Depression Scale; MoCA, Montreal Cognitive Assessment; PD, Parkinson disease; PDQ-39, 39-item Parkinson Disease Questionnaire; PDSS, Parkinson's Disease Sleep Scale; TUG, timed up-and-go; UPDRS-III, Unified Parkinson's Disease Rating Scale Part III*.

### Inclusion and Exclusion Criteria

The study's inclusion and exclusion criteria are presented in Table 2. Eligible patients will be right-handed (as determined by the Edinburgh Handedness Inventory), with no history of significant neurological or psychiatric disorders, have normal or corrected-to-normal vision, and be aged 55–75 years. They will have clinical diagnoses of Hoehn and Yahr stages I–III idiopathic PD without a family history of parkinsonism and be able to stand unaided, walk without an assistive device, and provide informed consent.

**Table 2:** Inclusion and exclusion criteria.

**Inclusion**	**Exclusion**
Aged 55–75 years	Surgery (e.g., deep brain stimulation)
Right-handed	Family history of parkinsonism
Without neurological or psychiatric disorder	Montreal Cognitive Assessment score <26
Hoehn and Yahr stages I–III	Symptoms of impulse control disorder
Stand unassisted	Histories of epilepsy
Walk without an assistive device	Participate in other exercise interventions

The following exclusion criteria will be applied: prior surgery (e.g., deep brain stimulation), overt signs of cognitive impairment [Montreal Cognitive Assessment (MoCA) score <26], symptoms of impulse control disorder (which accelerate the stopping of initiated motor actions), history of epilepsy, and participation in any other behavioral or instructor-led exercise program during the study period.

### Sample Size

Given that there has not been any previous TMS study examining cortical connectivity in people with PD after a *Wu Qin Xi* exercise intervention, we estimated the required sample size based on the number of subjects included in a previous behavioral experimental study (29). A power analysis in G^*^power software indicated that 63 participants (21/group) will be required to demonstrate effects on primary and secondary outcomes with repeated-measures analyses of variance (ANOVAs) at a 0.05 significance level and 80% power. The observation of significant interhemispheric inhibition of motor cortex changes in a sample of elderly individuals (10/group) obtained with a similar TMS paradigm (30) supports the adequacy of the proposed sample size. With anticipation of a 20% attrition rate, we plan to recruit 90 subjects (30/group).

### Interventions

#### *Wu Qin* Xi Exercise

**Figure 2:**
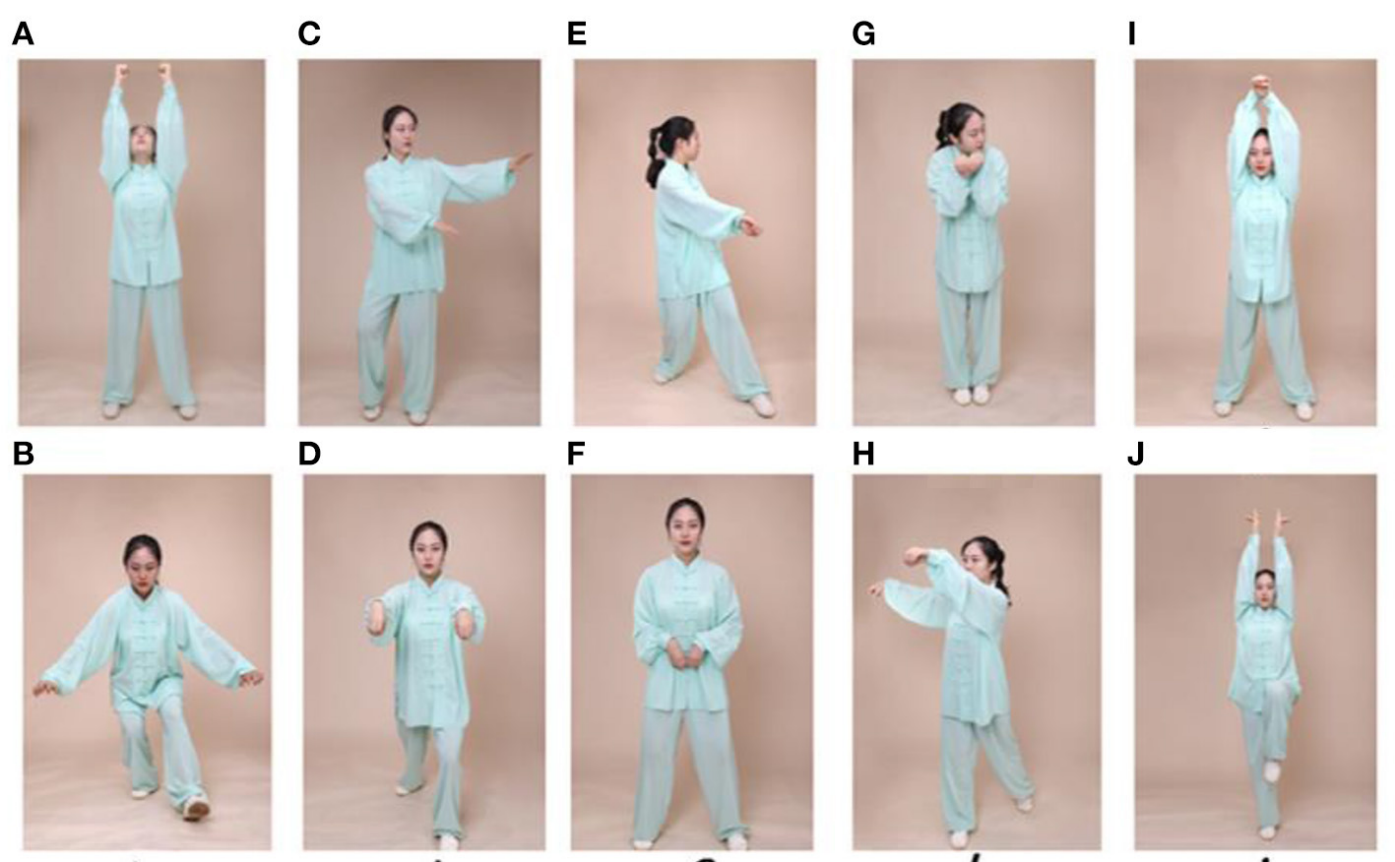
Exercise maneuvers. *Wu Qin Xi*, which translates to “five animals play,” is a commonly practiced traditional Chinese exercise. It involves full-body movements in which one imitates the movements and expressions of a tiger, a deer, a bear, a monkey, and a crane. Two moves for each animal as shown in this figure as follows: **(A,B)** tiger, **(C,D)** deer, **(E,F)** bear, **(G,H)** monkey, and **(I,J)** crane.

Participants in the experimental group will attend a 24-week *Wu Qin Xi* exercise course. The intervention will be implemented in a group format, with three 90-min sessions per week; each session will consist of a 10-min warm-up followed by 60 min of *Wu Qin Xi* exercises (with 10 min of rest within the exercise period), and a 10-min cooldown consisting of limb range-of-motion movements, sustained stretching, and relaxing (Figure 2). Detailed records of each person's performance will be collected at each intervention, and movements will be adjusted based on real-time practice so that they are individualized to the needs of each patient. During the sessions, participants' heart rates will be monitored with Polar-team 2 devices (Polar Electro, Finland) to enable progressive control of intensity. During the first 12 weeks, participants will be familiarized with the main components of the movements; subsequently, the focus will be on formal consistency of the movements, fluency of gait, posture, and balance. Participants will be guided to perform the entire range of movements that feels safe to them. After each weekly training session, participants will be encouraged to practice at home. Participants in the *Wu Qin Xi* exercise group will be asked to not perform other exercises at home during the intervention period.

#### Balance Exercise

PD participants in the balance exercise control group will attend a supervised balance exercise program of the same frequency, duration, and session length as the *Wu Qin Xi* groups. The format will be modeled after the experimental exercise format, including a 10-min warm-up, a 60-min exercise period with a 10-min break after the first 30 min, and 10-min cooldown (limb range-of-motion movements, sustained stretching, and relaxing). The balance training will consist of: (1) static balancing on unstable surfaces with the maintenance of postural control and progression to weight shifting; (2) dynamic balancing with postural control while standing and performing upper-limb and trunk movements; (3) balance strategy development with a focus on the hips while maintaining ankle balance, including stepping in different directions under interference; (4) and adaptation to varying bases of support and standing in a narrow space and on an uneven surface (31, 32).

#### Control Condition

Healthy control participants will maintain their normal work and daily routines, with no exercise intervention. They will attend a 90-min health lecture once a month on exercise and healthy eating habits for older adults.

### Evaluations

#### Transcranial Magnetic Stimulation Protocol

##### Electromyographic Recording

Surface electromyograms will be recorded from the right first dorsal interosseous (FDI) muscle with 9-mm-diameter Ag-AgCl surface electrodes. The active and reference electrodes will be placed over the stomach muscle and metacarpophalangeal joint, respectively. The signal will be amplified (1,000×), bandpass filtered (2–2.5 kHz; Intronix Technologies Model), digitized at 5 kHz by an analog–digital interface (Micro1401; Cambridge Electronics Design, Cambridge, UK), and saved for off-line analysis.

##### Transcranial Magnetic Stimulation

TMS will be applied to the bilateral M1 with a figure-of-eight-shaped coil (7-cm external loop diameter) connected to two single-pulse monophasic stimulators (Magstim Co., Whitland, Dyfeld, UK). The M1 hotspot will be defined as the scalp location inducing the largest peak–peak motor-evoked potential (MEP) amplitude in the contralateral FDI muscle. The handle of the test stimulus (TS) coil will be positioned posteriorly, 30°–45° off the midsagittal line. TS_1mV_ will be defined as the lowest TMS intensity required to generate MEPs of 1 mV in the relaxed FDI muscle in at least five of 10 trials. The resting motor threshold was defined as the lowest TMS intensity required to generate MEPs of more than 50 μV in at least five out of 10 trials with the target muscle completely relaxed (33).

##### Dual-Site Paired-Pulse Transcranial Magnetic Stimulation

**Figure 3:**
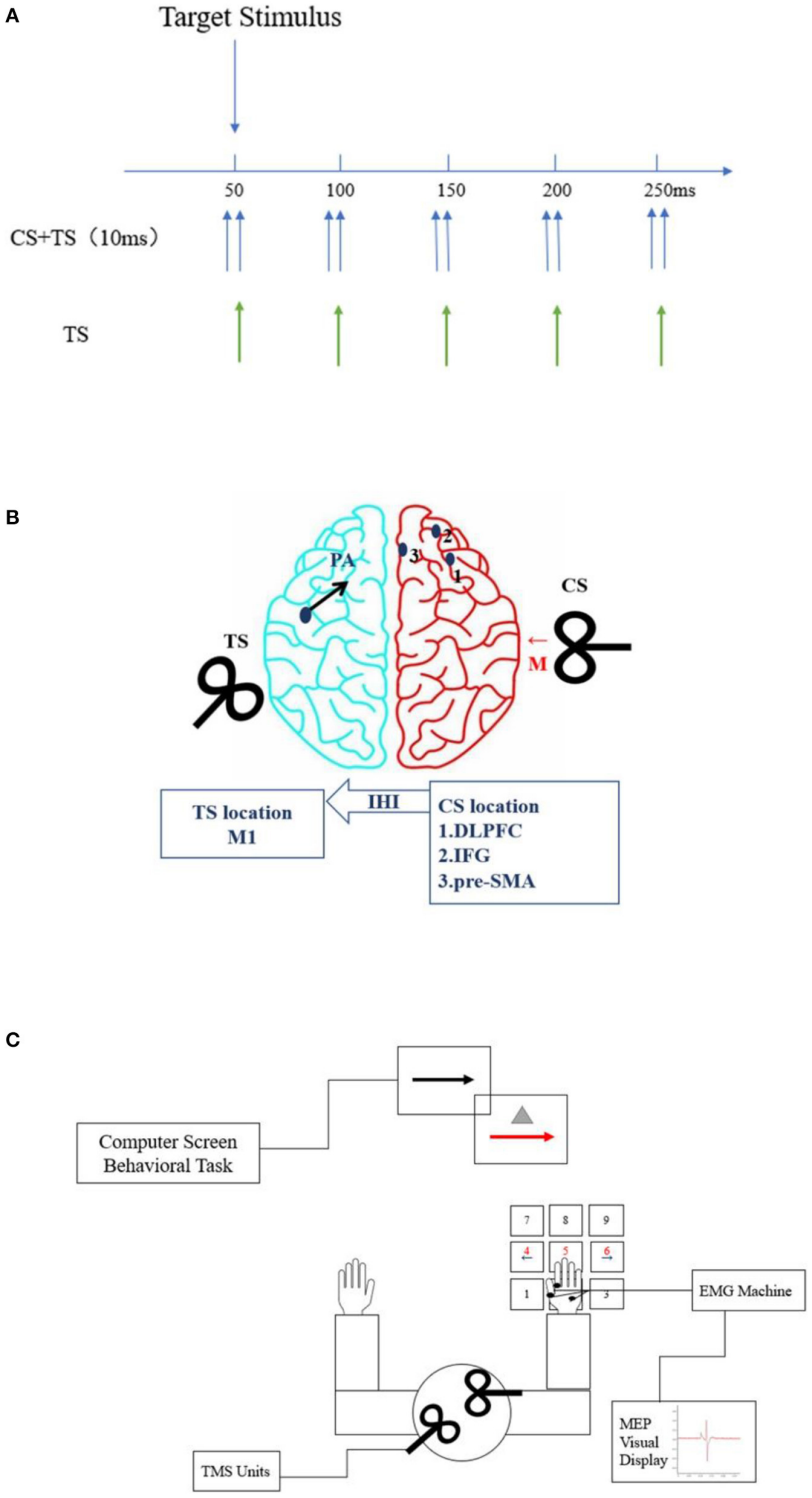
Placement of transcranial magnetic stimulation (TMS) coil sites and flow configuration of the study. **(A)** TMS setup. There was a 10-ms interval between the conditioning stimulus (CS) and test stimulus (TS). The stimulus signals of TS or TS + CS were applied at the time points of 50, 100, 150, 200, and 250 ms. **(B)** Stimulation sites (blue dots). Red and black arrows indicate CS and TS current directions, respectively. The CS coil (small figure-of-70 mm-coil) will be placed over the right hemisphere to induce medially (M) directed current in the brain (red arrow). The three blue dots represent the areas to be stimulated with the CS coil, namely, the dorsolateral prefrontal cortex (DLPFC) (1), right inferior frontal gyrus (rIFG) (2), and pre-supplementary motor area (pre-SMA) (3). The TS (regular figure-of-7 coil) will induce current in the posterior-to-anterior direction (blue arrow) and will be applied over the hand representation in the left hemisphere. **(C)** Schematic of go and stop trials with paired-pulse transcranial magnetic stimulation (ppTMS). The subjects will sit in front of the computer, with their right hand connected to the electromyogram (EMG), and perform keystroke response tasks. The coils will be placed in the target brain regions of each hemisphere for TMS stimulation. Motor-evoked potential (MEP) amplitudes will be displayed on the screen in real time.

To investigate changes in connectivity between the left M1 and right hemispheric response inhibition after long-term exercise training, we will apply ppTMS with two high-power Magstim 200 devices and two figure-of-eight coil sites (Figure 3A). Coil placement will be performed as in a similar hemispheric study (34) to avoid overlap. The smaller conditioning stimulus (CS) coil will be placed over the right hemisphere to induce medially directed current in the brain and will be used to stimulate the DLPFC, rIFG, and pre-SMA. The TS coil will be applied over the hand representation of the left hemisphere for induction of a posterior–anterior (PA) current in the brain. The CS intensity will be set to 110% of the resting motor threshold (RMT) based on the finding that a suprathreshold conditioning pulse can elicit functional interaction between the frontal lobe and M1 (19). The TS (M1) intensity will be set to evoke a resting MEP with the same TMS coil.

TMS assessments will be performed before and after the intervention and at a follow-up appointment 12 weeks after completion of the 24-week intervention period, with the subjects sitting relaxed in a chair (elbow, hip, and knee maintained at 90°–100°) in front of a computer monitor placed 75 cm in front of their eyes. Before the assessment, the subjects will receive instructions and practice the behavioral task for familiarization. They will be instructed to respond as quickly and accurately as possible to the arrow and not to delay their responses in anticipation of the stop signal. The zero time point will be set at the onset of the stimulus, and the TS will appear for 50, 100, 150, 200, and 250 ms [10 times per interstimulus interval (ISI) and 10 times for TS-alone trials; Figures 3B,C]. Participants will receive ppTMS stimulation during each ISI while pressing a button.

### Outcomes

#### Primary Outcomes

##### Behavioral Task: Response Inhibition Task

**Figure 4:**
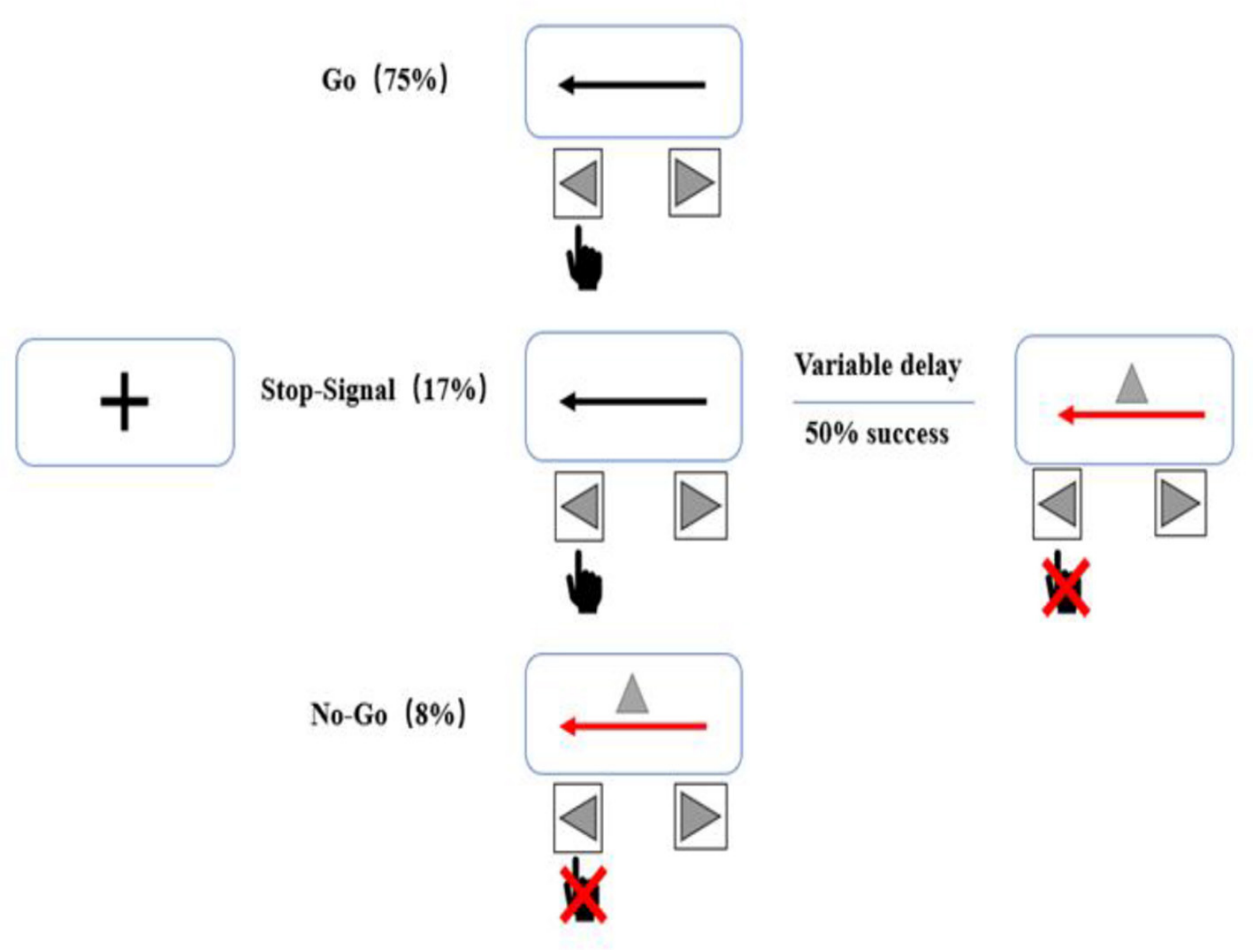
Stop behavioral task. The task will consist of randomly interleaved no-go and stop-signal trials, enabling us to examine both types of inhibition. Each session will include a total of 480 trials (75% go, 17% stop, and 8% no-go).

The task will consist of randomly interleaved no-go and stop-signal trials, enabling us to examine both types of inhibition (32). A total of 480 trials (75% go, 17% stop, and 8% no-go) will be administered (Figure 4). In go trials, subjects will respond to left- and right-pointing black arrows (displayed for 1,000 ms) by pressing corresponding buttons with the right index finger. In stop-signal trials, responses will be cued initially by left- and right-pointing black arrows, followed by a red arrow with a gray triangle (requiring non-response) after a stop-signal delay. The duration of the stop-signal delay will be varied from trial to trial using a step-up/down algorithm, with an initial duration of 250 ms to maintain 50% successful inhibition. In no-go trials, subjects will be required to make no response to the red arrow with the gray triangle (displayed for 1,000 ms) in a setup equivalent to a 0-ms stop-signal delay.

##### Cortical Connectivity

The ppTMS will be used to assess changes in cortical connectivity in specific brain regions associated with inhibitory control before and after the intervention and at the follow-up appointment.

### Secondary Outcomes

The subjects' mobility, balance, emotional state, motor symptoms, daily living ability, and sleep quality will be assessed using the following instruments: Timed up-and-go test (TUG), Hospital Anxiety and Depression Scale (HADS), Unified Parkinson's Disease Rating Scale Part III (UPDRS-III), Parkinson Disease Questionnaire (PDQ-39), and Parkinson Disease Sleep Scale (PDSS).

1. The TUG is a simple measure of mobility and motor ability in older adults. The total time, in s, needed for the subject to get up from a chair and walk 3 m at their usual walking pace, then turn around and walk back to the chair, and sit down (with his or her back against the seat) will be measured (35). The participants will be instructed to perform the test at a safe, self-selected pace.
2. The HADS is a 14-item clinical and research instrument widely used to screen patients with chronic illness for depression and anxiety symptoms in the past week and recommended for use in people with PD; due to potentially overlapping somatic symptoms, parkinsonian manifestations are not assessed with this scale (36, 37). It is composed of anxiety and depressive symptoms subscales. Clinically relevant thresholds for subscale and total scores are ≥8 and ≥15, respectively.
3. The UPDRS-III is currently recognized as the standard tool for the clinical evaluation of PD motor symptoms. Higher scores indicate greater PD motor sign severity.
4. The 39-item PDQ was used to assess health-related quality of life (exercise, daily living ability, emotional state, stigmatization, social support, state of consciousness, communication, and physical discomfort) in the previous month in people with PD. Each item is rated on a 5-point (0–4) ordinal scale, with higher scores indicating worse quality of life (38).
5. The 15-item PDSS was used to rate the severity of sleep disturbances in people with PD. Items inquire about daytime sleepiness, nocturia, and nocturnal sleep quality, restlessness, and psychiatric and movement symptoms. Patients rate the frequency or intensity of symptoms on a scale ranging from 0 (poor or frequent) to 10 (good or never), with a total possible score of 150 and higher scores indicating better sleep quality (39).

### Statistical Analysis

To explore the effects of *Wu Qin Xi* practice on inhibition of responses, between- and within-group differences in behavioral outcomes over time (pre-intervention, post-intervention, and 12-week follow-up) will be examined using mixed-model ANOVA. Peak–peak MEP amplitudes from the conditioned (CS and TS) TMS trials will be expressed as percentages of the mean peak–peak MEP amplitude from the unconditioned (TS only) test pulse. Values <100% and >100% will be taken to indicate inhibition and facilitation, respectively. Values will be reported as means ± standard errors of the mean. MEP values will be interpreted as representations of the influence of the DLPFC, pre-SMA, and rIFG on M1 under the given condition. Three-factor mixed-design repeated-measures ANOVA (three groups × three times × five ISIs) will be used to analyze the effect of *Wu Qin Xi* practice on the inhibitory control of brain network connections in people with PD. The MEP values will serve as the dependent variable, while time, group, and ISI will be treated as independent variables. Repeated-measures ANOVAs will be used to determine the effects of *Wu Qin Xi* practice on the secondary outcomes, with group and time as factors.

## Discussion

Exercise experience has been shown to improve inhibitory control (22), and aerobic exercise training has been found to promote processes related to inhibitory control selectively (40). However, primarily short-term, high-intensity exercise interventions have been reported to improve inhibition defects in people with PD (41). Studies showing that response inhibition overall is defective in people with PD have not examined the two distinct modes of reactive and proactive inhibition. Examination of the effects of long-term *Wu Qin Xi* practice on different inhibitory control modes in people with PD will improve our understanding of inhibitory disorders in this population and support the development of diversified rehabilitation training programs.

Long-term specialized exercise generates exercise experience-related changes in neural mechanisms (21–23) and enhances brain connectivity (42). Exercise can improve dopamine neurotransmission and increase local cerebral blood flow through the regulation of neuroplasticity in the cortical striatum (43, 44). Such changes may lead to enhancement of basal ganglia and cortical–thalamic neural circuits, which may, ultimately, lead to improvements in motor, non-motor, and cognitive behaviors in people with PD (45). Because dopamine loss in the frontal striatum is associated with cognitive deficits, the frontal region is considered an important reflex area for response inhibition control (46, 47). Thus, we will use ppTMS to detect changes in cortical connectivity after the *Wu Qin Xi* exercise intervention relative to baseline. We will explore the effects of long-term *Wu Qin Xi* exercise on functional changes coincident with cortico-cortical interaction connectivity changes with behavioral assessments of dyskinesia and executive ability for response inhibition control. To further clarify the effects of the long-term *Wu Qin Xi* intervention, the relationship between the two modes of response inhibition will be explored in relation to changes in prefrontal network connectivity. This neurological evidence will improve our understanding of the physiological basis of impulse control disorder in people with PD. If the present results provide evidence of beneficial effects of *Wu Qin Xi* on response inhibition ability in people with PD, then this study will provide neurological evidence favoring the use of a supplemental exercise-based clinical therapy.

This study will have several limitations. First, because we are recruiting only subjects with mild–moderate PD, our findings will not be generalizable to patients with advanced PD. Second, we did not plan to match subtypes of the disease, and the cohort may include patients with and without SWEDD (scans without evidence of a dopaminergic deficit). Studies have suggested that the inhibitory ability associated with abnormal activity in cerebello-thalamo-cortical circuits during movement in SWEDD cases may differ from those of patients with imaging evidence of a dopaminergic deficit. In future studies, we intend to consider patients with different PD subtypes comprehensively and to conduct experiments designed to delineate neural mechanisms (48). Finally, because the intervention will be apparent to the participants, the single-blind design may expose the study to risks of bias stemming from performance and evaluation, potentially leading to an overestimation of exercise effects.

## Ethics Statement

The studies involving human participants were reviewed and approved by Shanghai University of Sport research ethics committee. The patients/participants provided their written informed consent to participate in this study.

## Author Contributions

ZW contributed to the conceptualization, methodology, investigation, and writing the original draft. L-LZ contributed to the visualization, software, formal analysis, and validation. YW contributed to data curation. JZ contributed to the writing, review, and editing and project administration. KL contributed to the conceptualization, supervision, and funding acquisition. All authors contributed to the article and approved the submitted version.

## Conflict of Interest

The authors declare that the research was conducted in the absence of any commercial or financial relationships that could be construed as a potential conflict of interest.

## Abbreviations

DLPFC: dorsolateral prefrontal cortex
rIFG: right inferior frontal gyrus
pre-SMA: pre-supplementary motor area
ppTMS: paired-pulse transcranial magnetic stimulation
MEP: motor-evoked potential
RMT: rest motor threshold
TS: test stimulus
CS: conditioning stimulus
MoCA: Montreal Cognitive Assessment
UPDRS: Unified Parkinson's Disease Rating Scale
PDQ-39: 39-item self-report Parkinson Disease Questionnaire
PDSS: Parkinson's Disease Sleep Scale
HADS: Hospital Anxiety and Depression Scale
TUG: timed up-and-go
SWEDD: scans without evidence of dopaminergic deficit.

## References

[1] Braver TS. The variable nature of cognitive control: a dual-mechanisms framework shifting the emphasis to variability in cognitive control. Trends Cogn Sci. (2012) 16:106–13. 10.1016/j.tics.2011.12.010 22245618 PMC3289517

[2] Gauggel S Rieger M Feghoff TA. Inhibition of ongoing responses in patients with Parkinson's disease. J Neurol Neurosurg Psychiatry. (2004) 75:539–44. 10.1136/jnnp.2003.016469 15026491 PMC1739013

[3] Wylie SA van den Wildenberg WPM Ridderinkhif KR Bashore TR Power VD Manning CA. The effect of Parkinson's disease on interference control during action selection. Neuropsychologia. 47:145–57. 10.1016/j.neuropsychologia.2008.08.001 18761363 PMC4524676

[4] Aron AR. From reactive to proactive and selective control: developing a richer model for stopping inappropriate responses. Biol Psychiatry. (2011) 69:e55–68. 10.1016/j.biopsych.2010.07.024 20932513 PMC3039712

[5] Di Caprio V Modugno N Mancini C Olivola E Mirabella G. Early-stage Parkinson's patients show selective impairment in reactive but not proactive inhibition. Mov Disord. (2020) 35:409–18. 10.1002/mds.27920 31755149

[6] Aron AR Behrens TE Smith S Frank MJ Poldrack RA. Triangulating a cognitive control network using diffusion-weighted Magnetic Resonance Imaging (MRI) and functional MRI. J Neurosci. (2007) 27:3743–52. 10.1523/JNEUROSCI.0519-07.2007 17409238 PMC6672420

[7] Coxon JP van Impe A Wenderoth N Swinnen SP. Aging and inhibitory control of action: cortico-subthalamic connection strength predicts stopping performance. J Neurosci. (2012) 32:8401–12. 10.1523/JNEUROSCI.6360-11.2012 22699920 PMC6703643

[8] Rioult-Pedotti MS Friedman D Donoghue JP. Learning-induced LTP in neocortex. Science. (2000) 290:533–6. 10.1126/science.290.5491.533 11039938

[9] Fjell AM Sneve MH Grydeland H Storsve AB Walhovd KB. The disconnected brain and executive function decline in aging. Cereb Cortex. (2017) 27:2303–17. 10.1093/cercor/bhw082 27073220

[10] Stuckenschneider T Askew CD Menêses AL Baake R Weber J Schneider S. The effect of different exercise modes on domain-specific cognitive function in patients suffering from Parkinson's disease: a systematic review of randomized controlled trials. J Parkinson's Dis. (2019) 9:73–95. 10.3233/JPD-181484 30741688

[11] Garavan H Ross TJ Stein EA. Right hemispheric dominance of inhibitory control: an event-related functional MRI study. Proc Natl Acad Sci USA. (1999) 96:8301–6. 10.1073/pnas.96.14.8301 10393989 PMC22229

[12] Duchesne C Lungu O Nadeau A Robillard ME Boré A Bobeuf F. Enhancing both motor and cognitive functioning in Parkinson's disease: Aerobic exercise as a rehabilitative intervention. Brain Cogn. (2015) 99:68–77. 10.1016/j.bandc.2015.07.005 26263381

[13] Van Rooij SJH Rademaker AR Kennis M Vink M Kahn RS Geuze E. Impaired right inferior frontal gyrus response to contextual cues in male veterans with PTSD during response inhibition. J Psychiatry Neurosci. (2014) 39:330–8. 10.1503/jpn.130223 24886789 PMC4160362

[14] Ponzo V Picazio S Benussi A di Lorenzo F Brusa L Caltagirone C. Altered inhibitory interaction among inferior frontal and motor cortex in l-dopa-induced dyskinesias. Mov Disord. (2016) 31:755–9. 10.1002/mds.26520 26861941

[15] Albares M Thobois S Favre E Broussolle E Polo G Domenech P. Interaction of noradrenergic pharmacological manipulation and subthalamic stimulation on movement initiation control in Parkinson's disease. Brain Stimul. (2015) 8:27–35. 10.1016/j.brs.2014.09.002 25284704

[16] Van der Kolk NM de Vries NM Kessels RPC Joosten H Zwinderman AH Post B. Effectiveness of home-based and remotely supervised aerobic exercise in Parkinson's disease: a double-blind, randomized controlled trial. Lancet Neurol. (2019) 18:998–1008. 10.1016/S1474-4422(19)30285-6 31521532

[17] Guo Y Xu M Wei Z Hu Q Chen Y Yan J. Beneficial effects of qigong wuqinxi in the improvement of health condition, prevention, and treatment of chronic diseases: evidence from a systematic review. Evid Based Comp Altern Med. (2018). 2018:1–40. 10.1155/2018/3235950 30473716 PMC6220394

[18] Zhang F Bai YH Zhang J. The influence of “wuqinxi” exercises on the lumbosacral multifidus. J Phys Ther Sci. (2014) 26:881–4. 10.1589/jpts.26.881 25013288 PMC4085213

[19] Wang Y Cao N Lin Y Chen R Zhang J. Hemispheric differences in functional interactions between the dorsal lateral prefrontal cortex and ipsilateral motor cortex. Front Hum Neurosci. (2020) 14:1–6. 10.3389/fnhum.2020.00202 32581747 PMC7283611

[20] Van den Wildenberg WPM Burle B Vidal F van der Molen MW Ridderinkhof KR Hasbroucq T. Mechanisms and dynamics of cortical motor inhibition in the stop-signal paradigm: a TMS study. J Cogn Neurosci. (2010) 22:225–39. 10.1162/jocn.2009.21248 19400674

[21] Di Russo F Taddei F Apnile T Spinelli D. Neural correlates of fast stimulus discrimination and response selection in top-level fencers. Neurosci Lett. (2006) 408:113–8. 10.1016/j.neulet.2006.08.085 17018246

[22] Bianco V di Russo F Perri RL Berchicci M. Different proactive and reactive action control in fencers' and boxers' brain. Neuroscience. (2017) 343:260–8. 10.1016/j.neuroscience.2016.12.006 28003155

[23] Zhang S Tsai SJ Hu S Xu J Chao HH Calhoun VD. Independent component analysis of functional networks for response inhibition: inter-subject variation in stop signal reaction time. Hum Brain Mapp. (2015) 36:3289–302. 10.1002/hbm.22819 26089095 PMC4545723

[24] Reithler J Peters JC Sack AT. Multimodal transcranial magnetic stimulation: using concurrent neuroimaging to reveal the neural network dynamics of noninvasive brain stimulation. Prog Neurobiol. (2011) 94:149–65. 10.1016/j.pneurobio.2011.04.004 21527312

[25] Chou YH Hickey PT Sundman M Song AW Chen NK. Effects of repetitive transcranial magnetic stimulation on motor symptoms in Parkinson disease: a systematic review and meta-analysis. JAMA Neurol. (2015) 72:432–40. 10.1001/jamaneurol.2014.4380 25686212 PMC4425190

[26] Ni Z Gunraj C Nelson AJ Yeh IJ Castillo G Hoque T. Two phases of interhemispheric inhibition between motor related cortical areas and the primary motor cortex in human. Cereb Cortex. (2009) 19:1654–65. 10.1093/cercor/bhn201 19015374

[27] Rothwell JC. Using transcranial magnetic stimulation methods to probe connectivity between motor areas of the brain. Hum Mov Sci. (2011) 30:906–15. 10.1016/j.humov.2010.07.007 21056490

[28] Wilhelm E Quoilin C Petitjean C Duque J. A double-coil TMS method to assess corticospinal excitability changes at a near-simultaneous time in the two hands during movement preparation. Front Hum Neurosci. (2016) 10:1–11. 10.3389/fnhum.2016.00088 27014020 PMC4779885

[29] Wang T Xiao G Li Z Jie K Shen M Jiang Y. Wuqinxi exercise improves hand dexterity in patients with Parkinson's disease. Evid Based Comp Altern Med. (2020) 2020. 10.1155/2020/8352176 33178323 PMC7644302

[30] Wischnewski M Kowalski GM Rink F Belagaje SR Haut MW Hobbs G. Demand on skillfulness modulates interhemispheric inhibition of motor cortices. J Neurophysiol. (2016) 115:2803–13. 10.1152/jn.01076.2015 26961108 PMC4922604

[31] Atterbury EM Welman KE. Balance training in individuals with Parkinson's disease: therapist-supervised vs. home-based exercise programme. Gait Post. (2017) 55:138–44. 10.1016/j.gaitpost.2017.04.006 28445854

[32] Li Z Zhuang J Jiang Y Xiao G Jie K Wang T. Study protocol for a single-blind randomised controlled trial to evaluate the clinical effects of an Integrated Qigong exercise intervention on freezing of gait in Parkinson's disease. BMJ Open. (2019) 9:1–9. 10.1136/bmjopen-2018-028869 31515419 PMC6747653

[33] O'Shea J Sebastian C Boorman ED Johansen-Berg H Rushworth MFS. Functional specificity of human premotor-motor cortical interactions during action selection. Eur J Neurosci. (2007) 26:2085–95. 10.1111/j.1460-9568.2007.05795.x 17868374

[34] Ye Z Altena E Nombela C Housden CR Maxwell H Rittman T. Selective serotonin reuptake inhibition modulates response inhibition in Parkinson's disease. Brain. (2014) 137:1145–55. 10.1093/brain/awu032 24578545 PMC3959561

[35] Steffen T Seney M. Test-retest reliability and minimal detectable change on balance and ambulation tests, the 36-Item Short-Form Health Survey, and the Unified Parkinson Disease Rating Scale in people with parkinsonism. Phys Therapy. (2008) 88:733–46. 10.2522/ptj.20070214 18356292

[36] Eriksen S Bjørkløf GH Helvik AS Larsen M Engedal K. The validity of the hospital anxiety and depression scale and the geriatric depression scale-5 in home-dwelling old adults in Norway?. J Affect Disord. (2019) 256:380–5. 10.1016/j.jad.2019.05.049 31212233

[37] Rodriguez-Blazquez C Frades-Payo B Forjaz MJ de Pedro-Cuesta J Martinez-Martin P Aguilar M. Psychometric attributes of the hospital anxiety and depression scale in Parkinson's disease. Mov Disord. (2009) 24:519–25. 10.1002/mds.22321 19177496

[38] Opara JA Brola W Leonardi M Błaszczyk B. Quality of life in Parkinson's disease. J Med Life. (2012) 5:375–81. 10.1002/gps.2520 23346238 PMC3539848

[39] Xu Jichao NI Xiushi. Evaluation progress of testing and evaluation tool on quality of life of Parkinson's Disease. Chin Gen Pract. (2017) 20:1398–402. 10.3969/j.issn.1007-9572.2017.11.025

[40] Smith PJ Blumenthal JA Hoffman BM Cooper H Strauman TA Welsh-Bohmer K. Aerobic exercise and neurocognitive performance: a meta-analytic review of randomized controlled trials. Psychosomat Med. (2010) 72:239–52. 10.1097/PSY.0b013e3181d14633 20223924 PMC2897704

[41] Caciula MC Horvat M Tomporowski PD Nocera J. The effects of exercise frequency on executive function in individuals with Parkinson's disease. Mental Health Phys Act. (2016) 10:18–24. 10.1016/j.mhpa.2016.04.001

[42] Burdette JH Laurienti PJ Espeland MA Morgan A Telesford Q Vechlekar CD. Using network science to evaluate exercise-associated brain changes in older adults. Front Aging Neurosci. (2010) 2:1–10. 10.3389/fnagi.2010.00023 20589103 PMC2893375

[43] Petzinger GM Fisher BE Akopian G Holschneider DP Wood R Walsh JP. The role of exercise in facilitating basal ganglia function in Parkinson's disease. Neurodegen Dis Manag. (2011) 1:157–70. 10.2217/nmt.11.16 23805167 PMC3691073

[44] Petzinger GM Holschneider DP Fisher BE McEwen S Kintz N Halliday M. The effects of exercise on dopamine neurotransmission in Parkinson's disease: targeting neuroplasticity to modulate basal ganglia circuitry. Brain Plast. (2016) 1:29–39. 10.3233/BPL-150021 26512345 PMC4621077

[45] Fiorelli CM Ciolac EG Simieli L Silva FA Fernandes B Christofoletti G. Differential acute effect of high-intensity interval or continuous moderate exercise on cognition in individuals with Parkinson's disease. J Phys Act Health. (2019) 16:157–64. 10.1123/jpah.2018-0189 30626260

[46] Kehagia AA Barker RA Robbins TW. Neuropsychological and clinical heterogeneity of cognitive impairment and dementia in patients with Parkinson's disease. Lancet Neurol. (2010) 9:1200–13. 10.1016/S1474-4422(10)70212-X 20880750

[47] Jellinger KA. Cognitive impairment and dementia in Parkinson's Disease. Eur J Neurol. (2010) 17:e64. 10.1111/j.1468-1331.2010.03024.x

[48] Schirinzi T di Lorenzo F Ponzo V Palmieri MG Bentivoglio AR Schillaci O. Mild cerebello-thalamo-cortical impairment in patients with normal dopaminergic scans (SWEDD). Parkinson Relat Disord. (2016) 28:23–8. 10.1016/j.parkreldis.2016.03.023 27170027

